# The Physiological Anatomy and Physiology of Man

**Published:** 1857-07

**Authors:** 


ART. IX.—*The Physiological Anatomy and Physiology of Man.* By ROBERT BENTLEY TODD, M.D., F.R.S., Fellow of the College of Physicians, and Physician to King's College Hospital; and WILLIAM BOWMAN, F.R.S., Fellow of the College of Surgeons, Surgeon to King's College Hospital and The Royal London Ophthalmic Hospital; late Professor of Physiology and General and Morbid Anatomy in King's College, London. Republished by Blanchard & Lea, Philadelphia, complete in one volume, 8vo. pp. 926. With two hundred and ninety-eight Illustrations, 1857.

AFTER nearly fourteen years of not unremitted but severe labor, upon the part of the distinguished authors, this great work is at length brought to completion. The successive parts which have appeared from time to time, each immediately received the sanction of all true lovers of the science of histology, and long before the last was ready for the press, the work was quoted everywhere as the standard authority upon the various subjects treated of. We cannot therefore look upon the book as a new one, except so far as the concluding chapters are concerned, and all that we can at present say in regard to these, is that they fully bear out the high expectations that were based upon the former parts. We hope before long to return to this subject, for if there is a scientific book in the English language deserving our commendation, and an attempt to set forth its excellent qualities, in a way which shall lead some few, at least, to its appreciative perusal, it is the work in question.

And here we desire also to return our sincere thanks to the enterprising publishers, for furnishing the profession of this country with a book of such merit, at a comparatively trifling cost, and without, in the language of a well-known Boston reviewer, an "ascaris in the form of an appendix," by an American editor.

